# Flexible Electrochemical Platform Coupled with In Situ Prepared Synthetic Receptors for Sensitive Detection of Bisphenol A

**DOI:** 10.3390/bios12121076

**Published:** 2022-11-25

**Authors:** Chen-Yan Xu, Kang-Ping Ning, Zheng Wang, Yao Yao, Qin Xu, Xiao-Ya Hu

**Affiliations:** School of Chemistry and Chemical Engineering, Yangzhou University, Yangzhou 225002, China

**Keywords:** molecular imprinting, electrochemical, flexible, Bisphenol A

## Abstract

A flexible electrochemical sensor based on the carbon felt (CF) functionalized with Bisphenol A (BPA) synthetic receptors was developed. The artificial Bisphenol A receptors were grafted on the CF by a simple thermal polymerization molecular imprinting process. Fourier-transform infrared spectroscopy (FTIR), scanning electron microscopy (SEM) and electrochemical characterizations were used to analyze the receptors. Characterization results demonstrated that the Bisphenol A synthetic receptors successfully formed on the CFs surface. Because the synthetic receptor and the porous CFs were successfully combined, the sensor displayed a better current response once Bisphenol A was identified. The sensor’s linear range was determined to be from 0.5 to 8.0 nM and 10.0 to 300.0 nM, with a detection limit of 0.36 nM. Even after being bent and stretched repeatedly, the electrode’s performance was unaffected, demonstrating the robustness, adaptability and viability of installing the sensor on flat or curved surfaces for on-site detection. The designed electrochemical sensor has been used successfully to identify Bisphenol A in milk samples with satisfactory results. This work provided a promising platform for the design of implantable, portable and miniaturized sensors.

## 1. Introduction

Endocrine disruptors (EDC) are exogenous chemicals that disrupt the human endocrine system and increase the risk of diabetes, obesity, cardiovascular disease and prostate cancer [1]. Bisphenol A (2,2-bis (4-hydroxyphenyl) propane, BPA) has been functionalized as a kind of EDC to disturb the human endocrine system due to its structural resemblance to estrogen. However, Bisphenol A is a precursor material for the synthesis of epoxy resins, polycarbonate and polysulfone resins [2]. It is frequently present in plastic products, the inner coating of canned food and food packaging bags. Long-term storage and heat treatment can lead to the penetration of Bisphenol A into food from packaging materials [3,4]. This has raised concerns about food safety [5]. Therefore, the analysis and detection of Bisphenol A are particularly important.

Several analytical methods have been reported for the detection of Bisphenol A, including high-performance liquid chromatography (HPLC) [6], liquid chromatography-mass spectrometry (LC-MS) [7], gas chromatography-mass spectrometry (GC-MS) [8], spectrophotometry [9] and enzyme-linked immunosorbent assay (ELISA) [10]. However, the chromatography and mass spectrometry-based methods are expensive, time-consuming and require complex sample pretreatment. ELISA method is readily available from many manufacturers, but the preparation of antibodies is labor-intensive and expensive. ELISA method also suffers from the instability of biomolecules and short signal stability [11]. Therefore, the development of new methods with simplicity, stability and sensitivity that can detect trace amounts of Bisphenol A residue has become a major research challenge.

Electrochemical analysis technology with low cost, fast response speed and high sensitivity is a promising option for the development of inexpensive, simple and convenient Bisphenol A sensors [12,13,14]. Bisphenol A has two phenolic hydroxyl groups, which can be oxidized easily on the electrode surface [15]. This character makes it possible to identify Bisphenol A using straightforward electrochemical techniques. It is crucial to choose a material to enhance the performance of the electrochemical sensor when an electrochemical approach is put into practice. Currently, several materials, such as carbon nanomaterials and transition metal oxides, have been utilized as electrode modifiers to improve the electrochemical sensitivity for Bisphenol A detection [16,17,18]. However, the oxidation products of Bisphenol A would produce non-conductive polymers on the electrode surface, deactivating the electrode surface and preventing further reactions [19]. When Bisphenol A was first recognized and accumulated on the electrode surface, the selectivity and sensitivity of the electrochemical sensor could be improved [20].

**Figure 1:**
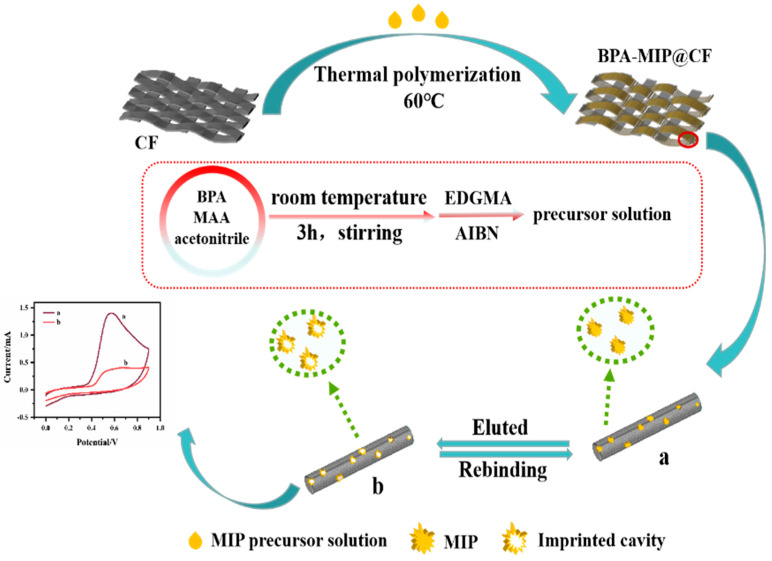
Schematic illustration of the preparation and application of MIP@CF sensor for the detection of BPA.

In recent years, synthetic receptors have been widely used in sensing platforms for recognition and accumulation. Molecularly imprinted polymers (MIPs) have become a research hotspot due to their superior selectivity over natural receptors, increased physicochemical stability and better selectivity [21,22]. Hamed E M et al. have summarized the different production methods and the varied types of sensors that employed MIPs for Bisphenol A detection [23]. Nanomaterials with large surface areas are always modified on the rigid electrodes to construct the electrochemical sensors [24]. However, the growing demand for portable and wearable electronics demands is beyond the capacity of rigid electrodes. It is important and necessary to build flexible sensors with good sensitivity and selectivity. CFs have received a lot of interest as a flexible electrode material due to their rich porosity structure and effective adsorption. Compared with conventional rigid electrodes, CF electrodes can be simply regenerated by ultrasonic cleaning [25]. The CF electrode can further be modified to offer a larger specific surface area, more active sites and better recognition ability [26]. In this work, a molecularly imprinted polymer layer was deliberately modified on the surface of a CF electrode by a simple in situ thermal polymerization process to fabricate a novel and sensitive electrochemical sensor for the detection of Bisphenol A [27]. Figure 1 depicts the preparation and application of the MIP@CF sensor for the detection of Bisphenol A. The molecularly imprinted precursor solution was created using the ideal molar ratio of Bisphenol A to methacrylic acid (MAA) and then was thermally polymerized on the surface of the CF. After the release of the template molecule Bisphenol A, the imprinted cavity of Bisphenol A remained on the MIP@CF electrode as the layer with selectivity. The imprinted cavity reidentified the Bisphenol A and provided an electrochemical response signal during the detecting procedure. Cyclic voltammetry (CV) was utilized to evaluate the electro-oxidation behaviors of the identified Bisphenol A, and differential pulse voltammetry (DPV) was employed to quantify Bisphenol A in real samples.

## 2. Experimental

### 2.1. Reagents and Apparatus

Graphite CFs, 2 mm thick, were purchased from Tianjin Carbon Factory (Tianjin, China). Acetonitrile (MW:41.05), ethanol (MW:46.07), methacrylic acid (MAA, MW: 86.09), Bisphenol A (MW:228.29), protocatechuic acid (PCA, MW:154.1), ascorbic acid (AA, MW:176.12), catechol (CC, MW:110.11) and hydroquinone (HQ, MW:110.11) with analytically purity were all purchased from Sinopharm Chemical Reagent Co., Ltd. (Shanghai, China). Ethylene glycol dimethacrylate (EGDMA, MW:198.22) was bought from Aladdin Industrial Corporation (Shanghai, China). Azobisisobutyronitrile (AIBN, MW:164.21) was obtained from Shanghai Shisi Hewei Chemical Co., Ltd. (Shanghai, China). All the reagents were of analytical grade and were used as received without further purification.

GeminiSEM 300 (German Carl Zeiss Co., Carl, Germany) was used to characterize the morphologies of the materials. The molecular structures of CF, Bisphenol A, Bisphenol A–MIP@CF and MIP@CF were checked and compared using a Cary 610/670 FTIR microscope (Varian, Palo Alto, CA, USA). The pH of solutions was adjusted using PHS-25 pH meter (Shanghai INESA Scientific Instrument Co., Ltd., Shanghai, China). A traditional three-electrode system was used for all the electrochemical experiments on a CHI-1040C workstation (Chenhua, Shanghai, China), using CF, MIP@CF and NIP@CF as working electrodes (working area = 1 cm^2^), a platinum wire as the counter electrode and an Ag/AgCl electrode as the reference electrode, respectively.

### 2.2. Preparation of MIP or NIP Modified Electrodes

The CFs were cut into 1.0 cm × 1.5 cm before they were ultrasonically cleaned with ethanol and deionized water for 15 min, respectively, then they were dried in a vacuum oven at 60 °C for 2 h for further use.

The MIP precursor solution was prepared by dissolving template Bisphenol A (0.5 mmol) and functional monomer MAA (2.5 mmol) in 12.5 mL of acetonitrile solution, stirring at room temperature for 3 h to make a homogeneous mixture. Then, 10 mmol of crosslinking agent EGDMA and 40 mg of initiator AIBN were added in turn, and the mixture was further stirred for 10 min, followed by bubbling with nitrogen for 20 min. Finally, the mixture was heated in a water bath at 60 °C for 30 min to form a pre-polymerized solution [28,29].

To prepare MIP–modified electrodes, the CFs were saturated with a pre-polymerized solution (400 μL) before being put on an electric heating table at 60 °C for in situ thermal polymerizations for 4 h. The obtained electrodes were named Bisphenol A–MIP@CF before the elution operation. Lastly, the BPA–MIP@CF electrode was immersed in acetic acid/methanol (1:9, V:V) eluent until the template molecule was fully removed. The resulting electrode was named MIP@CF. For comparison, NIP@CF electrodes were fabricated by the same procedure, except that the template molecule Bisphenol A was not added to the precursor solution during the polymerization process.

### 2.3. Bisphenol A Electrochemical Measurement

The incubation solution was a mixture of acetonitrile and PBS with a volume ratio of 1:9 and Bisphenol A with various concentrations. The MIP@CF was incubated at room temperature for a period of time in the incubation solution. Then the obtained electrode was taken out, thoroughly washed with PBS three times, and then immersed in a blank PBS solution for electrochemical analysis. The difference of current in MIP@CF electrode before and after capturing Bisphenol A reflects the concentrations of Bisphenol A.

### 2.4. Real Sample Analysis

Pure milk samples were purchased from a local supermarket. 2 mL of milk was diluted with 10 mL of PBS and centrifuged at 12,000 rpm for 15 min. The supernatant was collected and centrifuged again. Then, a certain amount of supernatant was mixed with PBS for electrochemical analysis. A certain amount of Bisphenol A standard solutions were added to the milk samples to determine the recovery rates of the assay.

## 3. Results and Discussions

### 3.1. Characterization of CF, Bisphenol A–MIP@CF, MIP@CF

**Figure 2:**
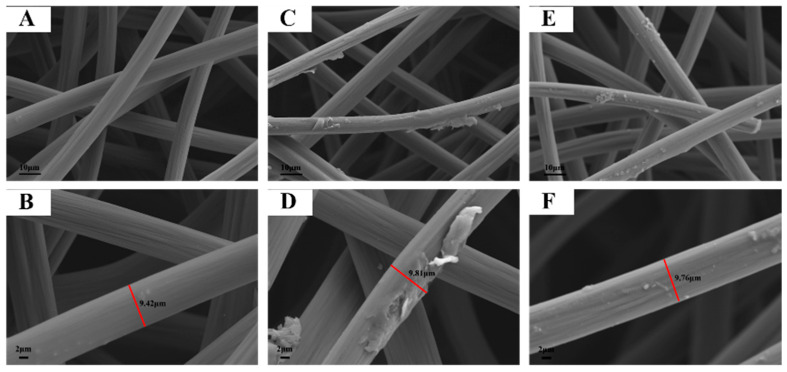
SEM images of bare CF (**A**,**B**), BPA–MIP@CF (**C**,**D**) and MIP@CF (**E**,**F**).

The surface morphologies of CF, Bisphenol A–MIP@CF and MIP@CF were analyzed and characterized by SEM. It was observed that the CF was composed of cross-linked networks (Figure 2A). The huge voids between carbon fibers were conductive to the transport of the incubation solution during the recognition process, while the cross-linked fibers were beneficial to the electron transfer to promote the electrocatalytic oxidation of Bisphenol A on the electrode [30,31,32]. In addition, there were abundant parallel cracks on the surface of carbon fiber (Figure 2B), and this roughness was beneficial for surface modification [33]. After the CF was modified by molecular imprinting technology, the diameter of the fiber was increased from 9.42 μm to 9.81 μm. The fiber surface became rough, and some bulk substances were loaded (Figure 2C,D), which may be the synthesized Bisphenol A molecularly imprinted polymers. For the MIP@CF electrode, as shown in Figure 2E,F, it could be clearly observed that the surface blocks were reduced, and the diameter of the fiber was slightly decreased to 9.76 μm. The elution process removed the template from the polymers. During this process, the loosely adhered polymer agglomerates were removed from the surface [34,35].

**Figure 3:**
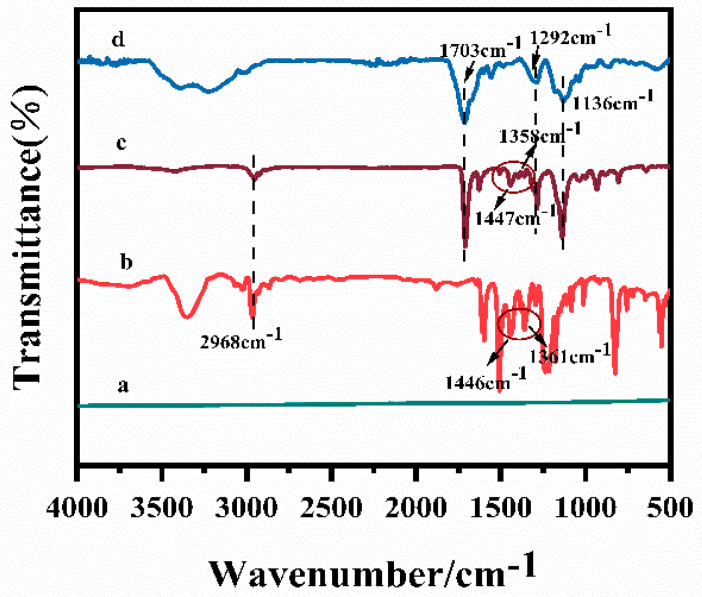
FTIR spectra of the CF (a), BPA (b), BPA–MIP@CF (c) and MIP@CF (d).

FTIR spectra of bare CF, Bisphenol A, BPA–MIP@CF and MIP@CF were studied and compared in Figure 3. The differences in these spectra confirmed the integration of new functional groups on the surface of the CF after imprinting. Compared with the bare CF (curve a) and Bisphenol A (curve b), the stretching vibration peak of the methylene group in the CF after Bisphenol A imprinting appeared at 2968 cm^−1^, and the characteristic peak of the benzene ring appeared at 1500 cm^−1^–1300 cm^−1^, which proved that the Bisphenol A molecule was successfully imprinted on the surface of the CF (curve c). No peak in the range of 1500 cm^−1^–1300 cm^−1^ was observed in this position of CF after elution of Bisphenol A, indicating that Bisphenol A has been successfully eluted from the electrode surface [36]. In addition, the characteristic absorption peaks at 1703 cm^−1^, 1292 cm^−1^ and 1136 cm^−1^ were observed from curves c and d, which were attributed to the C=O vibration peaks of carboxyl group in MAA and the symmetric and asymmetric stretching vibration peaks of C-O of ester group in EGDMA. The methylene peak, which was attributed to the C-H stretching of the methylene group of EGDMA and AIBN, also appears at 2998 cm^−1^ [37,38,39]. These results indicated that Bisphenol A had been successfully imprinted on the surface of CF, and a molecular imprint cavity was formed after elution.

### 3.2. Flexibility Tests of MIP@CF

**Figure 4:**
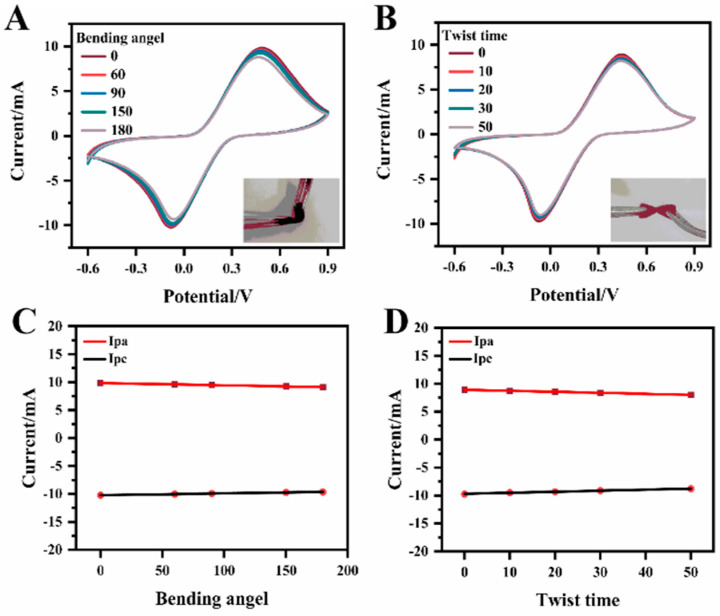
CVs of MIP@CF electrode with different bending angle (**A**) and twist time (**B**) in 5.0 mmol·L^−1^ K_4_[Fe(CN)_6_] with 0.1 mol·L^−1^ KCl at the scan rate of 50 mV·s^−1^; Relationship between the anodic (I_pa_) and cathodic peak (I_pc_) currents vs. bending angle (**C**) and twist time (**D**).

The MIP@CF electrodes express superior flexibility. CV images under different bending angles and twist times were studied. Figure 4A showed that the shapes of the CV curves were basically unchanged after the electrode bending angle was from 0 to 180°. In addition, CV curves remain almost unchanged after twisting 50 times (Figure 4B), indicating that bending and twisting did not affect the electrochemical performance of the electrode [40,41,42]. The insets of Figure 4A,B also confirmed the flexibility of the electrodes. Almost no change was observed in the oxidation and reduction peak current after bending and twisting (Figure 4C,D). The results showed that the electrode has good application potential in flexible electronics.

### 3.3. Electrochemical Behaviors of Different Modified Electrodes

**Figure 5:**
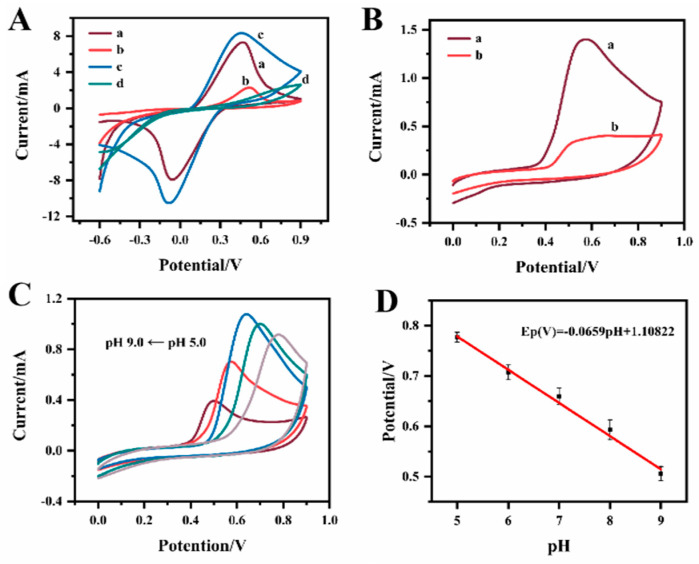
(**A**) CV responses of bare CF (a), BPA–MIP@CF (b), MIP@CF (c) and NIP@CF (d) in 5.0 mmol·L^−1^ K_4_[Fe(CN)_6_] with 0.1 mol·L^−1^ KCl at the scan rate of 50 mV·s^−1^; (**B**) CV curves of bare CF (b), MIP@CF (a) after recognizing 1.0 μM BPA; (**C**) CV responses of MIP@CF electrode at different pH (5.0, 6.0, 7.0, 8.0 and 9.0) after recognizing 1.0 μM BPA at the scan rate of 50 mV·s^−1^; (**D**) The relationship between the peak potentials and pH values.

The electrochemical behaviors of different electrodes were compared using the CV technique. Figure 5A showed the CV of the CF, BPA–MIP@CF, MIP@CF and NIP@CF in KCl solution containing 5 mM [Fe(CN)_6_]^3−/4−^. It can be seen from the figure that the bare CF electrode, without any modification, exhibits an obvious pair of well-separated redox peaks (curve a), indicating the good electron transfer ability of CF. Compared with the unmodified CF, the BPA–MIP@CF only showed an oxidation peak current at Epa of 605 mV. The redox peaks of [Fe(CN)_6_]^3−/4−^ disappeared (curve b). The polymer film on the CF inhibited the transfer of the [Fe(CN)_6_]^3−/4−^ to the electrode, so the redox peaks of [Fe(CN)_6_]^3−/4−^ were diminished. However, Bisphenol A imprinted in the polymer film could be oxidized at Epa of 605 mV [43]. The CV of NIP@CF supported this conclusion (curve d). No oxidation or reduction peak was observed on the NIP@CF. The formed non-conductive polymer layer on the CF hindered the electron transfer of [Fe(CN)_6_]^3−/4−^. The lack of the oxidation peak at around 605 mV also supported the fact that the peak was attributed to the oxidation of Bisphenol A [39]. The curve c in Figure 5A was the CV response of MIP@CF, which left the cavity on the polymer after the removal of Bisphenol A template. The redox peak of [Fe(CN)_6_]^3−/4−^ appeared again. After the elution of the template from the polymer, some cavities were left on the polymer. [Fe(CN)_6_]^3−/4−^ could diffuse through these cavities to the conductive CF surface, and electron transfer process was performed on the CF. The redox current on MIP@CF was slightly larger than that on the bared CF. This means that the extraction of template resulted in plenty of transport channels that were favorable to the redox reactions of [Fe(CN)_6_]^3−/4−^. The sensing performance of the MIP@CF for Bisphenol A detection was evaluated by comparing the oxidation current and oxidation potential of Bisphenol A on CF and MIP@CF. It can be seen from Figure 5B that Bisphenol A has obvious oxidation peak on both electrodes, and no corresponding reduction peak was found in the reverse scanning. This result indicates that the oxidation reaction of Bisphenol A on both electrodes was irreversible. Furthermore, the MIP@CF electrode (a) exhibited a significantly strong oxidation peak for Bisphenol A compared with bare CF (b). This was due to the specific recognition and accumulation of Bisphenol A by the imprinted sites [44]. The scan rate study is possible to understand whether the oxidation/reduction reaction mechanism of the analyte is controlled by adsorption or diffusion process. The CV responses of 1.0 μM of Bisphenol A on a MIP@CF were recorded at different scan rates (Figure S1). The oxidation peak current increased gradually with the increase in scan rate with a linear relationship in the range of 10–100 mVs^−1^. The corresponding linear regression equation was: Ip (mA) = 0.00065 + 0.0227 υ (mVs^−1^). This indicated that the oxidation of Bisphenol A on the MIP@CF was a typical adsorption-controlled process [45]. These results indicated that the MIP@CF can be used as an electrochemical tool for sensitive detection of Bisphenol A.

The pH value of the solution was an important parameter because it affected the oxidation potential of Bisphenol A. As shown in Figure 5C,D, the peak potential of Bisphenol A shifted negatively when the pH value increased from 5.0 to 9.0. The linear relationship between the oxidation peak potential (Ep) and pH was: Ep(V) = −0.0659·pH + 1.10822 (R^2^ = 0.994). The slope of 66 mV/pH is close to the theoretical value of 59 mV/pH unit. The results showed that the oxidation reaction of Bisphenol A on the MIP@CF was accompanied by an equal amount of proton and electron transfer processes [46].

### 3.4. Parameters Optimization for Sensor Preparation

**Figure 6:**
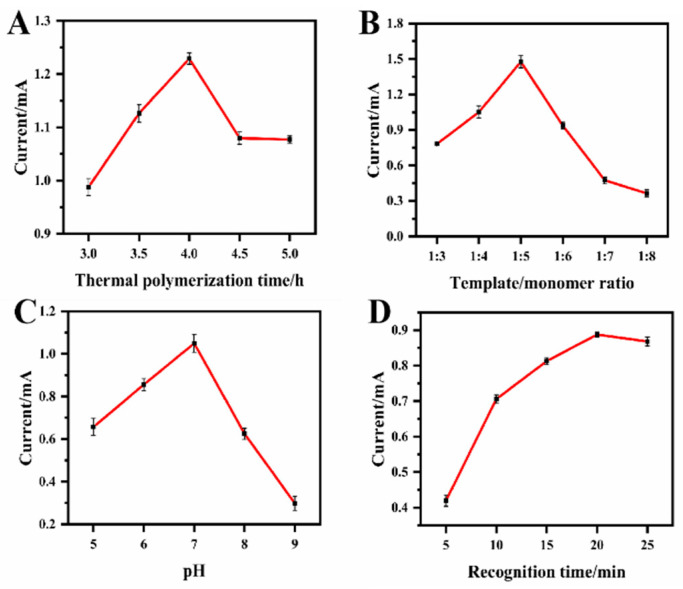
Plots of the oxidation currents of BPA versus (**A**) template/monomer ratio; (**B**) thermal polymerization time; (**C**) pH value of the detection solution and (**D**) elution time after the accumulation of 1.0 μM BPA.

In order to obtain the optimal sensing performance of the MIP@CF sensor for Bisphenol A detection, various experimental parameters were studied and optimized. Since the molar ratio of the template molecule and the functional monomer played an important role in the structure and rebinding affinity of the polymer, the molar ratio of Bisphenol A and MAA was adjusted from 1:3 to 1:8, and the corresponding oxidation peak currents were measured respectively. As shown in Figure 6A, the oxidation current value was the largest when the Bisphenol A: MAA ratio was 1:5. On the one hand, when the molar ratio of MAA continued to increase, excessive MAA monomer hindered the recognition site of Bisphenol A and resulted in a decrease in oxidation current. On the other hand, when the proportion of template molecule Bisphenol A was too high, insufficient number of monomers led to insufficient polymerization, and it was difficult to form effective imprinting binding sites. The obtained MIP film would have lower imprinting efficiency [47]. Therefore, the optimal molar ratio of template molecule to functional monomer was set as 1:5 in the preparation of the MIP@CF sensor.

The thermal polymerization time would influence the formation of the MIP layer on the CF surface and thus affect the performance of the MIP@CF sensor. It can be seen from Figure 6B that the oxidation current value gradually increases as the polymerization time increases and reaches the maximum current value at 4 h. However, the current value decreases as time continues to increase. This showed that the imprinted site had reached saturation after 4 h of thermal polymerization. Therefore, 4 h served as the optimal polymerization time for subsequent experiments.

Figure 6C shows that the current value of the oxidation peak is influenced by the pH of the detection solution. The peak current is the largest when pH 7.0 PBS is used. At higher pH, the decrease in peak current may be due to the electrostatic repulsion of the negatively charged anionic Bisphenol A to the sensor surface [48]. Therefore, a pH of 7.0 PBS was subsequently selected for the detection of Bisphenol A.

Figure 6D shows the effect of incubation time on the oxidative current after the MIP@CF sensor was incubated in the recognition solution, which contained Bisphenol A at different times. The oxidation current value gradually increased with the prolongation of incubation time and reached the maximum value after 20 min. So 20 min was selected as the optimum recognition time for Bisphenol A detection.

### 3.5. Sensing-Performance of MIP@CF in Bisphenol A Detection

**Figure 7:**
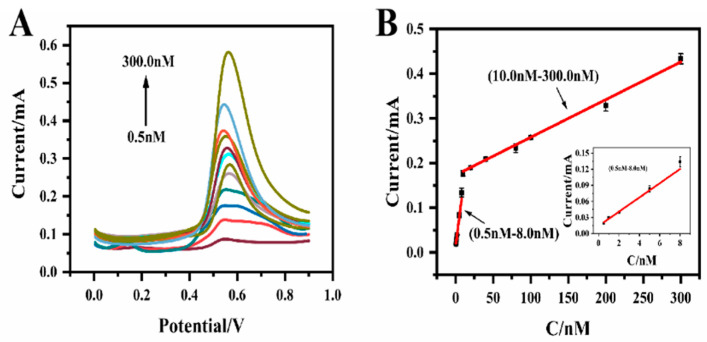
(**A**) DPV responses of BPA in blank pH 7.0 PBS after the recognition of different concentrations of BPA; (**B**) Calibration curves for BPA in the concentration ranges of 0.5 nM to 8.0 nM and 10.0 nM to 300.0 nM. The inset plot represented the calibration curve in the concentration ranges of 0.5 nM to 8.0 nM.

The analytical performance of MIP@CF for Bisphenol A detection was evaluated by differential voltammetry pulse method (DPV). Figure 7A shows the response of different concentrations of Bisphenol A on the MIP@CF. It was observed that the peak currents increased linearly with the concentration of Bisphenol A. Figure 7B showed the linear relationship between DPV peak currents and Bisphenol A concentrations. Two calibration curves could be seen in the figure. In 0.5 nM to 8.0 nM range, the regression equation was: I_P_(μA) = 13.28·C (nM) + 14.18. Bisphenol A was easily converted into its oxidation products on the electrode surface in this lower concentration range and had a high detection sensitivity. In 10.0 nM to 300.0 nM range, the regression equation was: I_P_(μA) = 0.843·C (μM) + 173.47. The identification of the target and the diffusion of reaction products at high concentrations require more oxidation reaction time than when the concentration is low. Moreover, the surface of the MIP@CF electrode will be contaminated by high concentration of oxidation products. These facts result in relatively low detection sensitivity. The limit of detection (LOD) was as low as 0.36 nM based on $\frac{3s_{y/x}}{b}$, where $s_{y/x}$ was the standard deviation of y-intercepts of the regression line and b was the slope of the calibration curve in the low concentration range of Bisphenol A [49].

Compared with other methods shown in Table 1, this work exhibits better sensing performance in terms of wider linear range and lower detection limit.

### 3.6. Selectivity

**Figure 8:**
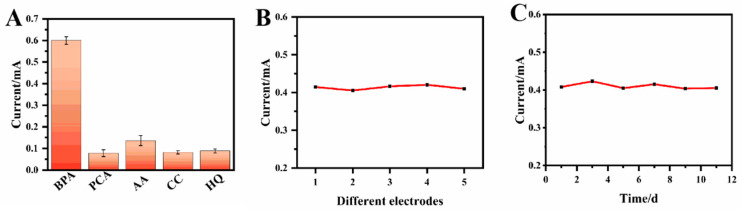
Investigating the selectivity of the MIP@CF sensor for 0.5 μM of Bisphenol A or 5.0 μM of the interferences (**A**), reproducibility (**B**) and stability (**C**) of MIP@CF electrodes for 5.0 μM of BPA.

The selectivity of the method was evaluated by measuring the DPV responses of different interfering substances on the MIP@CF sensors. The electrodes were incubated in 0.5 μM of Bisphenol A or 5.0 μM of the interferences, including protocatechuic acid (PCA), ascorbic acid (AA), catechol (CC) and hydroquinone (HQ). The DPV responses of these compounds are shown in Figure S2. There was almost no oxidation peak of these interfering substances near the specific oxidation peak potential of Bisphenol A, and the high response signal to Bisphenol A verified the high selectivity of the sensor. Figure 8A shows the current responses of Bisphenol A and the interfering substances at about 0.6 V. It was shown that the presence of 10 times interferences does not have significant effects on the oxidation peak of Bisphenol A. The presence of the special binding site regions in MIP polymers ensures the specific recognition of Bisphenol A at the imprinted site through the hydrogen bonds and Van der Waals force and makes the sensor have good selectivity. The special oxidation peak potential also contributed to the good selectivity of the sensor for Bisphenol A detection.

### 3.7. Reproducibility and Stability of the Sensor

The reproducibility of the MIP@CF sensor for 0.5 μM of Bisphenol A was measured by measuring the current responses of five different modified electrodes to Bisphenol A under the optimal conditions. Figure 8B,C showed the result that the relative standard deviation (RSD) of the five electrodes was 3.7%, indicating the excellent reproducibility of the sensor. The prepared electrodes were stored in a refrigerator at 4 °C for 12 days, and the response of the electrodes to the same concentration of Bisphenol A was measured every 2 days to determine its stability. The results showed that the relative standard deviation (RSD) of the method was 4.1%, indicating the acceptable stability of the sensor.

### 3.8. Real Samples Analysis

To verify the analytical potential of the sensor in the detection of real samples, the sensor was applied to detect Bisphenol A in pure milk samples. Bisphenol A was not detected in pure milk samples. Therefore, the recovery experiments were performed. A certain amount of Bisphenol A was added to the pure milk samples, and the recoveries were tested (Figure S3). The analysis results are shown in Table 2. It could be seen that the recovery of Bisphenol A is between 91.26–112%. The results showed that MIP@CF can be used as a novel electrochemical sensor for the detection of Bisphenol A in real samples.

## 4. Conclusions

In this work, a Bisphenol A synthetic receptor was deliberately modified on a flexible CF by a simple thermal polymerization process to construct a Bisphenol A electrochemical sensor. The receptor and the flexible CF work together to give the sensor excellent sensitivity, stability, selectivity and flexibility. This sensor showed linear DPV current responses to Bisphenol A from 0.5 to 8.0 nM and 10.0 to 300.0 nM. A good recovery result was obtained when this sensor was applied for the detection of Bisphenol A in milk samples. This work provides an effective way for the development of versatile electrochemical sensing platforms for the diagnosis of EDCs.

## Figures and Tables

**Table 1:** Performance comparison of the prepared sensor for Bisphenol A detection with other sensors.

Electrode	Method	Linear Range (μM)	LOD (nM)	Reference
MWCNTs-βCD/SPCE	LSV	0.125–2/2–30	13.7	[50]
CTAB/MIL-101(Cr)	DPV	0.02–0.35	10	[51]
NiFe_2_O_4_/SPCE	DPV	0.02–12.5	6	[52]
AgNPs-EG	SWV	5–100	230	[19]
AuNDs@CNFs/SPE	DPV	0.01–50	5	[53]
GO-MWCNT-βCD/SPE	LSV	0.05–5/5–30	6	[43]
BZPY/MCPE	DPV	2–18	29	[54]
MIP@CF	DPV	0.0005–0.008/0.01–0.3	0.36	This work

**Table 2:** Determination of BPA in real samples.

Sample	Original (nM)	Added (nM)	Found (nM)	Recovery (%)	RSD (%)
Milk	0.00	1.00	1.12	112	3.96
	0.00	50.00	51.28	102.5	4.16
	0.00	200.00	182.50	91.26	3.54

## Data Availability

Not applicable.

## Supplementary Materials

The following supporting information can be downloaded at: https://www.mdpi.com/article/10.3390/bios12121076/s1. Figure S1. (A) CVs of 1.0 μmol·L^−1^ BPA at MIP@CF electrode at different scan rates; (B) The oxi-dation peak currents of 1.0 μmol·L^−1^ BPA against scan rates; Figure S2. DPV responses of the MIP@CF electrode in pH 7.0 PBS solution after the recognition of 0.5 μM of Bisphenol A or 5.0 μM of protocatechuic acid (PCA), ascorbic acid (AA), catechol (CC), and hydroquinone (HQ); Figure S3. Electrochemical response of MIP@CF for milk sample analysis.

## Abbreviations

BPA: Bisphenol A; FTIR: Fourier-transform infrared spectroscopy; SEM: Scanning electron microscope; S/N: signal to noise; EDC: Endocrine disruptor; HPLC: high-performance liquid chromatography; LC-MS: liquid chromatography-mass spectrometry; GC-MS: gas chromatography-mass spectrometry; ELISA: enzyme-linked immunosorbent assay; MIP: Molecularly imprinted polymers; CF: carbon felt; MAA: methacrylic acid; DPV: differential voltammetry pulse method; EGDMA: ethylene glycol dimethacrylate; NIP: non-molecularly imprinted polymers; AIBN: Azobisisobutyronitrile; PBS: phosphate buffered saline; MW: molecular weight.
