# Supplementary material for: Flexible Electrochemical Platform Coupled with In Situ Prepared Synthetic Receptors for Sensitive Detection of Bisphenol A
Source: Biosensors (Basel). 2022 Nov 25;12(12):1076. doi: 10.3390/bios12121076 (PMC9775942; doi:10.3390/bios12121076)
Supplement: Supplementary file 1 [file biosensors-12-01076-s001.zip › biosensors-2003426-supplementary.pdf]

biosensors

Supporting Information

Flexible Electrochemical Platform Coupled with In Situ Prepared Synthetic Receptors for Sensitive Detection of Bisphenol A

Chen-Yan Xu, Kang-Ping Ning, Zheng Wang, Yao Yao, Qin Xu * and Xiao-Ya Hu

School of Chemistry and Chemical Engineering, Yangzhou University, Yangzhou 225002, China
* Correspondence: xuqin@yzu.edu.cn

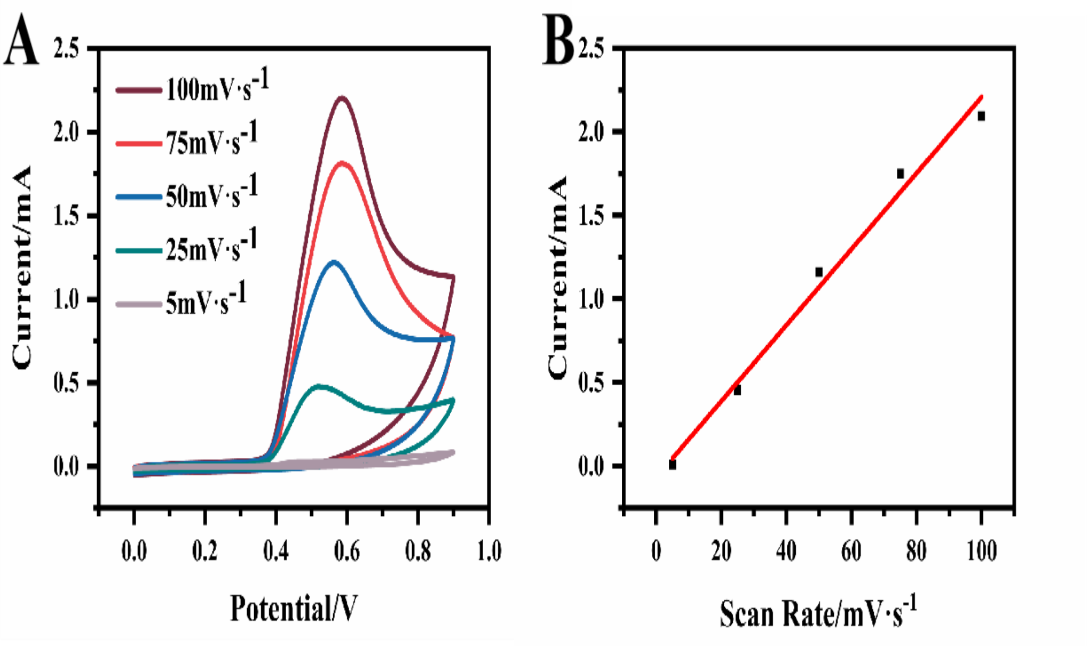

Figure S1. (**A**) CVs of 1.0 μmol·L^{-1} BPA at MIP@CF electrode at different scan rates; (**B**) The oxidation peak currents of 1.0 μmol·L^{-1} BPA against scan rates.

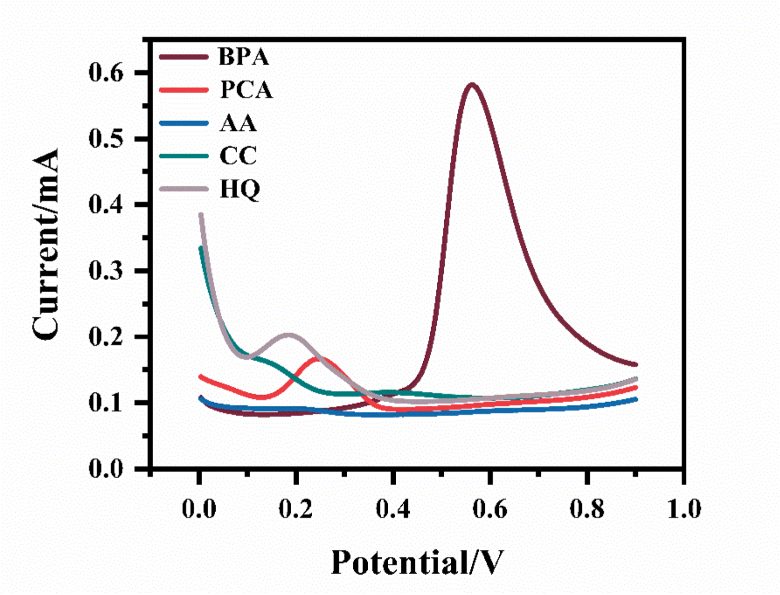

Figure S2. DPV responses of the MIP@CF electrode in pH 7.0 PBS solution after the recognition of 0.5 μM of Bisphenol A or 5.0 μM of protocatechuic acid (PCA), ascorbic acid (AA), catechol (CC), and hydroquinone (HQ).

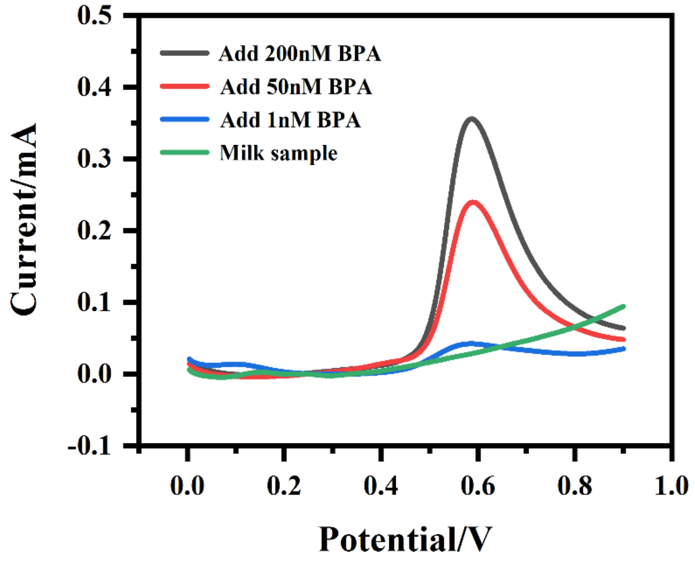

Figure S3. Electrochemical response of MIP@CF for milk sample analysis.
